# A Novel ‘*Candidatus* Liberibacter asiaticus’-Encoded Sec-Dependent Secretory Protein Suppresses Programmed Cell Death in *Nicotiana benthamiana*

**DOI:** 10.3390/ijms20225802

**Published:** 2019-11-18

**Authors:** Chao Zhang, Xuefeng Wang, Xuelu Liu, Yanyan Fan, Yongqiang Zhang, Xueping Zhou, Weimin Li

**Affiliations:** 1 Institute of Plant Protection, Chinese Academy of Agricultural Sciences, Beijing 100094, China; zhangchao01@caas.cn; 2 Biotechnology Research Institute, Chinese Academy of Agricultural Sciences, Beijing 100081, China; lxl1443485975@163.com (X.L.); f17701338173@163.com (Y.F.); zhangyongqiang@caas.cn (Y.Z.); 3 Citrus Research Institute, Southwest University, Chongqing 400712, China; wangxuefeng@cric.cn; 4 College of Life Science, Shandong Normal University, Jinan 250014, China

**Keywords:** *Candidatus* Liberibacter asiaticus, secreted protein, programmed cell death, suppression, Huanglongbing

## Abstract

‘*Candidatus* Liberibacter asiaticus’ (CLas) is one of the causal agents of citrus Huanglongbing (HLB), a bacterial disease of citrus trees that greatly reduces fruit yield and quality. CLas strains produce an array of currently uncharacterized Sec-dependent secretory proteins. In this study, the conserved chromosomally encoded protein CLIBASIA_03875 was identified as a novel Sec-dependent secreted protein. We show that CLIBASIA_03875 contains a putative Sec- secretion signal peptide (SP), a 29 amino acid residue located at the N-terminus, with a mature protein (m3875) of 22 amino acids found to localize in multiple subcellular components of the leaf epidermal cells of *Nicotiana benthamiana*. When overexpressed via a *Potato virus X* (PVX)-based expression vector in *N. benthamiana*, m3875 suppressed programmed cell death (PCD) and the H_2_O_2_ accumulation triggered by the pro-apoptotic mouse protein BAX and the *Phytophthora infestans* elicitin INF1. Overexpression also resulted in a phenotype of dwarfing, leaf deformation and mosaics, suggesting that m3875 has roles in plant immune response, growth, and development. Substitution mutagenesis of the charged amino acid (D7, R9, R11, and K22) with alanine within m3875 did not recover the phenotypes for PCD and normal growth. In addition, the transiently overexpressed m3875 regulated the transcriptional levels of *N. benthamiana* orthologs of *CNGCs* (cyclic nucleotide-gated channels), *BI-1* (Bax-inhibitor 1), and *WRKY33* that are involved in plant defense mechanisms. To our knowledge, m3875 is the first PCD suppressor identified from CLas. Studying the function of this protein provides insight as to how CLas attenuates the host immune responses to proliferate and cause Huanglongbing disease in citrus plants.

## 1. Introduction

Upon invasion of the host, phytopathogens produce numerous compounds (e.g., metabolites) to facilitate their survival in the host. These compounds are termed pathogen-associated molecular patterns (PAMPs), some of which are detected by host pattern recognition receptors (PRRs) localized in the cell membrane. The resulting interaction between the PAMPs and PRRs promotes (1) transcriptional reprogramming, (2) protein phosphorylation, (3) the activation of ion channels, (4) the production of reactive oxygen intermediates, (5) cell wall reinforcement, and (6) the accumulation of antimicrobial compounds. Collectively, these protective measures lead to PAMP-triggered immunity (PTI), the first inducible plant innate immunity [1,2]. To enable a compatible interaction, pathogens often suppress PTI by utilizing effector proteins. However, some disease resistance (*R*) genes in the plant cells may recognize these pathogen effectors, thereby stimulating the second layer of immunity, termed effector-triggered immunity (ETI). ETI is a strong local defense response that often culminates in hypersensitive response (HR). Therefore, this selective pressure may drive the evolution of mutants that either suppress ETI by acquiring additional effectors or avoid ETI entirely by shedding or diversifying the effectors that are recognized by the plant [1,3].

The past few decades have seen extensive efforts to characterize the defense-suppressing effectors from a broad range of pathogens, including viruses, bacteria, fungi, oomycetes, and nematodes [4,5,6,7,8]. Many Gram-negative bacterial pathogens, such as *Pseudomonas syringae*, *Ralstonia solanacearum*, and *Xanthomonas campestris*, have been found to deliver a complex cocktail of virulence effectors into host cells via the type III secretion system (T3SS). These effectors subvert the host ubiquitination system, directly modify host proteins, modulate host transcription, or perturb hormone signaling, thereby acting to suppress the host innate immune response [5,9,10].

‘*Candidatus* Liberibacter asiaticus’ (CLas) is a Gram-negative α-proteobacterium and is one of the presumptive causal agents of Huanglongbing (HLB, also known as citrus greening), the most devastating disease of citrus worldwide [11,12,13,14,15]. In nature, this bacterial pathogen is transmitted among the citrus plants by Asian citrus psyllid, a phloem-feeding insect widespread in most citrus-producing areas of Asia, Africa, and the Americas. After invading the citrus plants, CLas inhabits the phloem sieve elements and causes the rapid decline and ultimate death of entire trees [12,13,16].

To date, few citrus varieties have been identified that confer resistance to CLas [17,18], indicating the ability of this bacterium in suppressing the innate immune response of the citrus plants. It has been reported that two characterized CLas-encoded, non-classically secreted proteins, CLIBASIA_RS00445 and SC2_gp095, function as peroxidases that significantly inhibit the transcription of *RbohB*, a gatekeeper of H_2_O_2_-mediated defense signaling in *Nicotiana tabacum* [19,20]. Recently, CLIBASIA_RS00445 was also found to suppress oxylipin-mediated defense signaling in citrus [21]. In addition, the Sec-dependent secretory protein CLIBASIA_05315 was shown to physically interact with the citrus papain-like cysteine proteases (PLCPs), a group of defense regulators, to reduce the activity of the PLCPs [22]. The pieces of emerging evidence indicate that the CLas secreted proteins play critical roles in suppression of the host immune system.

CLas is an intracellular pathogen [14] that does not possess the T3SS, IV and VI secretion systems, the apparatuses commonly employed by Gram-negative bacteria to deliver effectors into host cells [23,24,25]. Instead, CLas has the full components of the Sec secretion machinery [26]. Mining of the CLas genome has resulted in the identification of 86 proteins that possess functional Sec-secretion signals and are potentially secreted into host cells via the Sec system [27]. In this study, we identified a new Sec-dependent secretory protein CLIBASIA_03875 (GenBank No. ACT57351.1), which is comprised of only 51 amino acids and includes a putative Sec-secretion signal peptide (SP) at the N-terminus of 29 amino acids. The mature form (C-terminal 22 amino acids, designated m3875) of CLIBASIA_03875 was found to suppress programmed cell death (PCD) triggered by both the pro-apoptotic mouse protein BAX (Genbank No. NP_031553) and the *Phytophthora infestans* elicitin INF1 (Genbank No. AAV92913). In addition, the overexpression of m3875 via a *Potato virus* X (PVX)-based vector [28] induced the phenotypes of dwarfing, leaf mottling, and deformation in *N. benthamiana*. To the best of our knowledge, this is the first PCD suppressor identified from CLas. Insights from these data may begin to explain how CLas utilizes secreted proteins to counteract the host immune responses.

## 2. Results

### 2.1. CLIBASIA_03875 Was Conserved among the CLas Strains

CLIBASIA_03875 was annotated in CLas psy62 [26] and Ishi-1 [29] and was a small putative chromosomal protein comprised of 51 amino acids. A BLAST search of the Genbank database showed that the coding sequence of CLIBASIA_03875 was present in all the available CLas genomes to date (Supplementary Figure S1) but was absent from ‘*Candidatus* Liberibacter africanus’ (CLaf) and ‘*Candidatus* Liberibacter americanus’ (CLam), the other two causal agents of HLB [14,30]. Therefore, CLIBASIA_03875 may be a conserved protein of the CLas strains.

### 2.2. CLIBASIA_03875 Was a Sec-Dependent Secretory Protein, and Its Mature Form Was Present in Multiple Subcellular Components of N. benthamiana Cells

**Figure 1:**
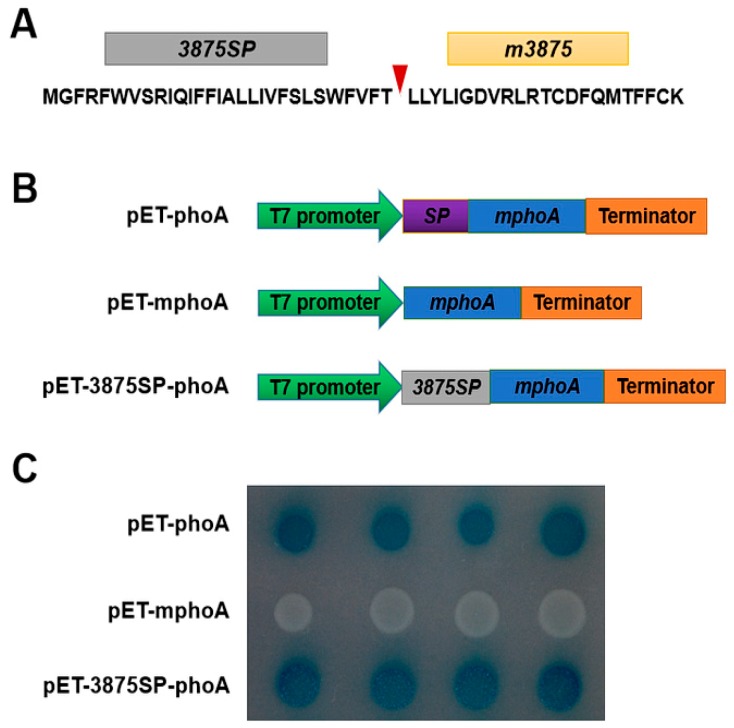
CLIBASIA_03875 is a Sec-dependent secretory protein. (**A**) Primary structure of CLIBASIA_03875. The triangle indicates the putative cleavage site. The putative signal peptide (SP) was designated 3875SP, and the mature form of CLIBASIA_03875 was designated m3875. (**B**) Diagrams of the prokaryotic expression cassettes for the *phoA* gene. pET-phoA harboring the full length *phoA* gene was used as a positive control, while pET-mphoA was a negative control that harbors *mphoA* lacking its native SP-encoding sequence. (**C**) 3875SP directed the extracytoplasmic export of the mPhoA moiety. After 6 h of incubation at 37 °C on LB media containing BCIP (90 μg/mL), IPTG (100 mM) and Na_2_HPO_4_ (75 mM), the *Escherichia coli* cells expressing mphoA remained colorless, but the cells carrying pET-3875SP-mphoA clearly turned blue, like those that harbored pET-phoA.

A bioinformatics analysis using PrediSi [31] revealed that CLIBASIA_03875 contained a putative signal protein (SP) (3875SP) corresponding to its N-terminal 29 amino acids (Figure 1A), which is basically consistent with a prediction based on SignalP [32] in a recent study [33]. To validate the extracytoplasmic transport signal function of 3875SP, its coding sequence was inserted into the pET- mphoA vector (Figure 1B) and was subjected to an *Escherichia coli* (*E. coli*) *phoA* gene fusion assay [34]. The cells expressing the fusion protein 3875SP-mphoA, like the positive control cells that harbored pET-phoA, turned dark blue after 6 h of incubation on indicator LB agar, while the *E. coli* cells that harbored pET- mphoA (the negative control) remained colorless even after 24 h of incubation (Figure 1C). Collectively, the in silico prediction and experimental data provide evidence that 3875SP directs the extracytoplasmic export of the mPhoA moiety in *E. coli*, indicating that CLIBASIA_03875 was a novel Sec-dependent secretory protein of CLas.

**Figure 2:**
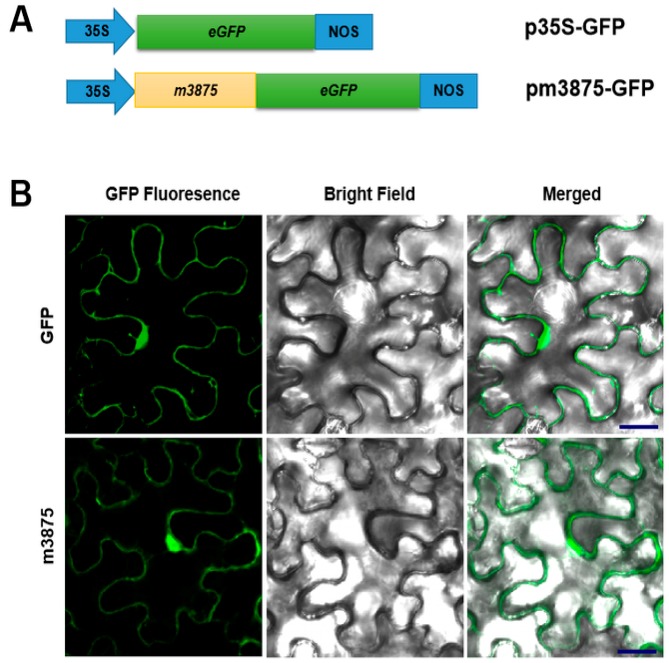
Subcellular localization of m3875 in *Nicotiana benthamiana*. (**A**) Diagrams of the expression cassettes for green fluorescent protein (GFP) and the fusion gene *m3875-GFP*. The CaMV 35S promoter is designated 35S, and the nopaline synthase terminator is designated NOS. (**B**) Fluorescence of the transiently expressed m3875-GFP in epidermal cells of the *N. benthamiana* leaves. p35S-GFP and pm3875-GFP were individually introduced into the five-leaf stage *N. benthamiana* plants grown at 25 °C via agroinfiltration, and the GFP fluorescence was viewed by confocal microscopy after 48 h post inoculation. Scale bars represent 20 µm.

Sec-dependent secretory proteins simultaneously undergo SP cleavage during extracytoplasmic translocation, yielding a mature protein [35]. Accordingly, after the removal of 3875SP, CLIBASIA_03875 would transition to its mature form, m3875. Since CLas is an intracellular pathogen [14], m3875 would be released directly into the host cells. To determine the distribution of m3875 in plant cells, a fusion protein m3875-GFP (Figure 2A) was expressed in *Nicotiana benthamiana* via *Agrobacterium*-mediated transient expression [36]. Using confocal microscopy, we observed that the green fluorescence of m3875-GFP, resembling that of free green fluorescent protein (GFP), was present in the nucleus, cytoplasm and cytoplasmic membrane (Figure 2B), indicating the multiple subcellular localization of m3875 in plant cells.

### 2.3. m3875 Suppressed Bax- and INF1-Triggered PCD in Nicotiana benthamiana

PCD functions in a variety of cellular processes, including plant immunity. A specialized form of PCD termed HR occurs in response to a pathogen and triggers rapid cell death to restrict the biotrophic/hemibiotrophic pathogens at the site of infection [37,38].

**Figure 3:**
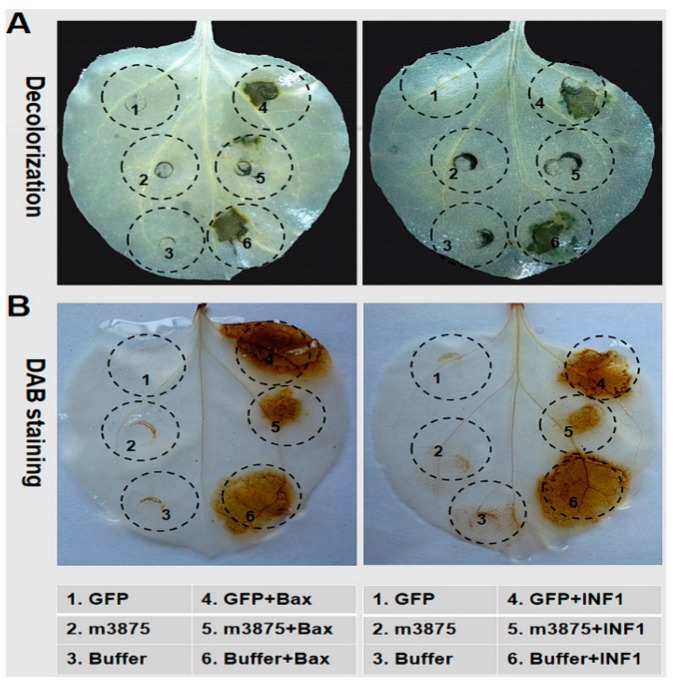
Suppression of PCD by m3875. (**A**) The m3875 suppressed both BAX- and INF1-triggered cell death in *Nicotiana benthamiana*. The *N. benthamiana* leaves were infiltrated with inoculation buffer or *Agrobacterium tumefaciens* cells expressing m3875 or green fluorescent protein (GFP), either alone or followed 24 h later with *A. tumefaciens* cells carrying the *BAX* or *INF1* gene. The leaves were subjected to decolorization with ethanol and visualized 5 days after *BAX*- or *INF1*-infiltration. (**B**) m3875 inhibited BAX- and INF1-induced H_2_O_2_ accumulation in *N. benthamiana*. Infiltration was performed as described above, and the leaves were detached at 2 days after *BAX*- or *INF1*-infiltration for 3,3′-diaminobenzidine (DAB) staining.

The mouse BAX and the PAMP INF1 of *Phytophthora infestans* are two well-known HR PCD inducers [39,40,41]. Based on a PVX-based expression system, both BAX and INF1 have been extensively utilized to identify the PCD suppressor from a range of phytopathogens in *N. benthamiana* [42,43,44,45,46]. With the same strategy, we found that m3875 completely suppressed both BAX- and INF1- triggered PCD (Figure 3A). The histochemical detection of H_2_O_2_, a critical reactive oxygen species (ROS) that contributes to plant cell death [47], revealed that BAX- and INF1-induced H_2_O_2_ accumulation were significantly inhibited by m3875 (Figure 3B). In contrast, the negative controls (infiltration buffer and GFP) did not suppress either cell death or the H_2_O_2_ accumulation triggered by BAX or INF1 (Figure 3A,B).

**Figure 4:**
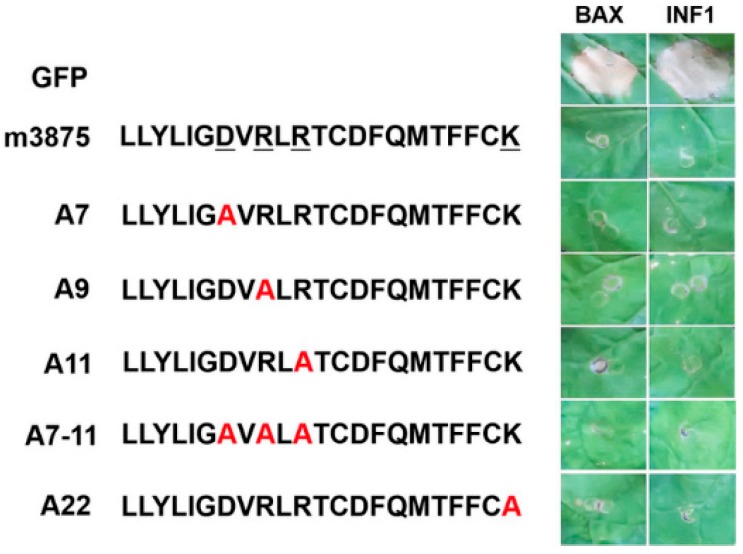
Effect of alanine point mutations on m3875-mediated PCD suppression. The charged amino acids, D^7^, R^9^, R^11^, and K^22^, within m3875 were individually substituted with alanine that is highlighted in read letter, resulting in A7, A9, A11, A7-11, and A22 (Left panel). Through agroinfiltration, the four m3875 mutants were transiently expressed in the *Nicotiana benthamiana* leaves 24 h prior to infiltration with *A. tumefaciens* cells harboring the *BAX* or *INF1* gene, and the photos were taken at 5 days post *BAX*- or *INF1*- infiltration (Right panel).

To identify the amino acids of m3875 involved in PCD suppression, the charged amino acids (D^7^, R^9^, R^11^ and K^22^) within the protein were individually or simultaneously substituted with the neutral amino acid alanine, resulting in mutants A7, A9, A11, A7-11 and A22 (Figure 4). As a result, all the mutants exhibited only a slightly reduced ability to suppress BAX- and INF1-mediated PCD (Figure 4 and Table 1), indicating the mild effects of D^7^, R^9^, R^11^ and K^22^ on PCD suppression. It was notable that the activity of A7-11, the mutant that harbored the simultaneous substitution of D^7^, R^9^ and R^11^ with alanine residues, remained nearly unchanged at suppressing PCD, comparable to that of the wild-type (WT) m3875 (Figure 4 and Table 1). Given the relatively small size (22 amino acids) of m3875, the data suggested the robustness of m3875 in the role of PCD suppression.

### 2.4. m3875 Interfered with the Development of Nicotiana benthamiana

**Figure 5:**
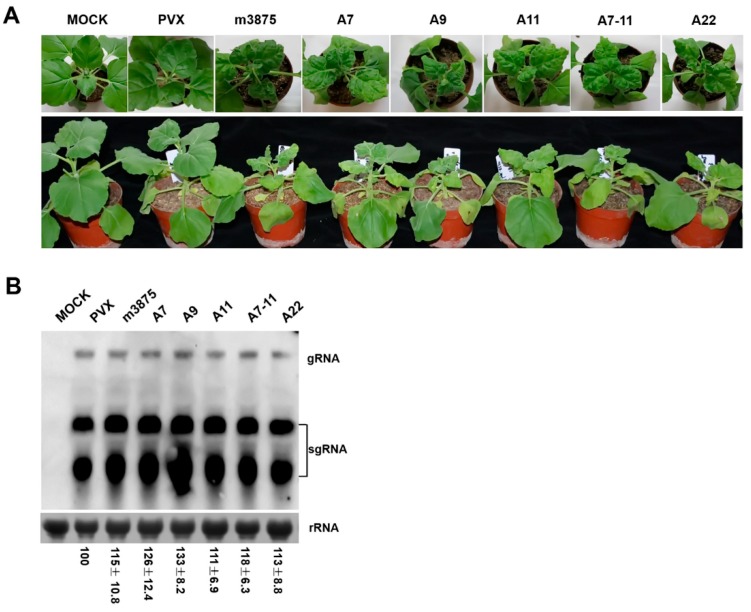
Characteristics of the PVX-m3875 hybrids in systemically infected *Nicotiana benthamiana* at 15 days post inoculation (dpi). (**A**) Systemic symptoms of PVX, PVX-m3875, PVX-A7, PVX-A9, PVX- A11, PVX-A7-11 and PVX-A22. The PVX-infected *N. benthamiana* plants showed little difference compared with the mock-inoculated *N. benthamiana*, while the plants infected with the PVX vector expressing m3875 or the mutants developed dwarfing as well as leaf deformation and mosaic. (**B**) Northern blot analysis of PVX RNA. Total RNA was extracted from the upper systemically infected leaves of a similar developmental stage. Bands that corresponded to the PVX viral RNAs are indicated, and rRNA stained with methylene blue is shown as a loading control. Numbers represent the means with the standard deviation (SD), indicating the relative abundance of the gRNA normalized to that of PVX. The experiment was repeated three times with similar results.

To further explore the biological significance of m3875 in planta, *A. tumefaciens* cells with pPVX- m3875 were infiltrated into the *N. benthamiana* seedlings (Figure 4), and *A. tumefaciens* cells harboring empty PVX-releasing pGR107 were included as a parallel control. Both the PVX- and PVX- m3875-infected plants initially induced systemic symptoms of leaf crinkling and veinal chlorosis at 5–6 days post inoculation (dpi). Continuous observations up to 15 dpi showed that the PVX-infected plants gradually recovered and were comparable with the healthy *N. benthamiana*. However, the plants infected with PVX-m3875 developed severe symptoms, including dwarfing, leaf deformation and mosaic (Figure 5A). Northern blot analysis detected a similar viral load of PVX and PVX-m3875 in the upper leaves of *N. benthamiana* (Figure 5B), indicating that m3875 had little impact on the multiplication of PVX, despite functioning as a PCD suppressor. Taken together, these data indicated that the severe symptoms displayed by PVX-m3875-infected plants were most likely caused by the heterologously expressed m3875, rather than the increased replication of PVX, suggesting that m3875 interfered with plant development.

Since amino acid changes had little effect on m3875-mediated PCD suppression (Figure 4), we questioned if the mutations disrupted the interference of m3875 in plant development. Using the same strategy as above, *A. tumefaciens* cells harboring pPVX-A7, pPVX-A9, pPVX-A11, pPVX- A7- 9- 11 or pPVX-A22 were infiltrated into *N. benthamiana*. All the mutants, including A7-11, induced symptoms similar to that of the WT m3875 (Figure 5A) and had little effect on the multiplication of PVX (Figure 5B), providing additional evidence for the robustness of m3875 in its associated roles.

### 2.5. m3875 Regulated Transcription of the Defense-Related Genes in Nicotiana benthamiana

**Figure 6:**
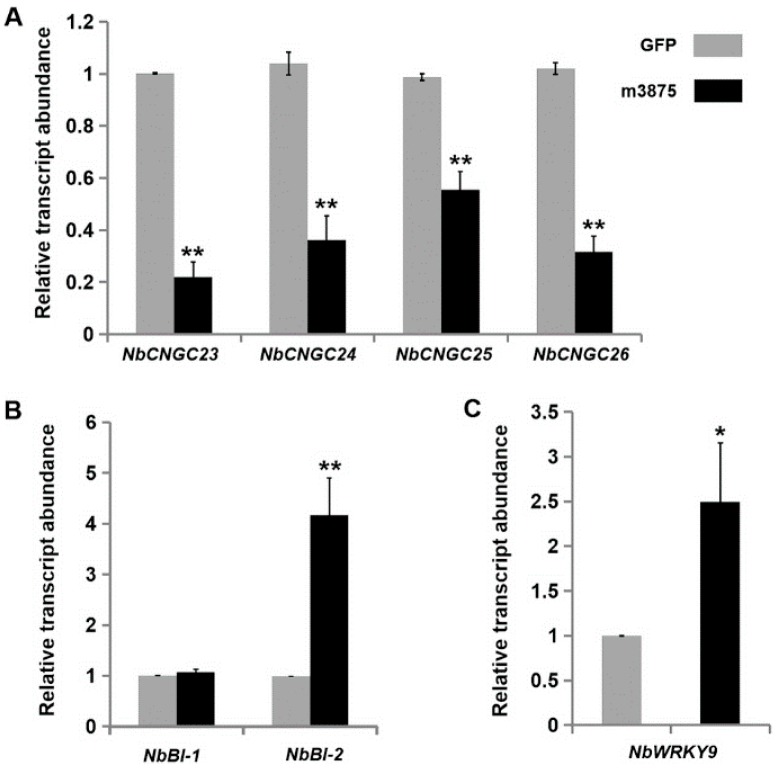
Effect of m3875 on the regulation of the defense-associated genes in *Nicotiana benthamiana*. Total RNA was extracted from the leaves that transiently expressed m3875 or GFP at 48 h post inoculation (hpi) and used to measure the relative expression levels of *NbCNGC23*, *NbCNGC24*, *NbCNGC25*, *NbCNGC26* (**A**), *NbBI-1*, *NbBI-2* (**B**), and *NbWRKY9* (**C**) using RT-qPCR. Three biological replicates (each consisting of three technical replicates) were performed. Expression levels of the indicated genes were individually normalized to the internal reference *Nbactin*. Bars represent the mean ± standard error, and asterisks denote significant differences according to a Student’s *t* test (** *p* < 0.01 and * *p* < 0.05).

Since m3875 suppressed the HR PCD formation, we then examined the transcriptional levels of the defense-related genes in *N. benthamiana* that transiently expressed m3875 and eGFP. Calcium is a universal secondary messenger that has been implicated in plant innate immunity [48]. In plants, Ca^2+^ transport across the plasma membranes is mediated by several families of ion channels, including cyclic nucleotide-gated channels (CNGCs) [49,50]. The plant CNGCs family is classified into five groups (I, II, III, IVa, and IVb) [51] with the members of group-IVb determined to be positive regulators of HR formation [52,53]. Thus, the transcripts of all four *CNGC* group-IVb members (*NbCNGC23*-*26*) in *N. benthamiana* were studied [54]. The expression of all the genes tested were significantly reduced in the *N. benthamiana* leaves transiently expressing m3875 compared with those expressing eGFP, further supporting the hypothesis that m3875 acts to suppress the host immune response (Figure 6A).

We next focused on Bax-inhibitor 1 (BI-1), a common cell death suppressor in plants, as well as animals [55]. In *N. benthamiana*, there are two *BI-1* homologs (designated *NbBI-1* and *NbBI-2*), which share ~84% identity [56]. Interestingly, m3875 dramatically enhanced the expression of *NbBI-2* but had only a minor effect on *NbBI-1* (Figure 6B). In addition, the transcriptional level of *NbWRKY9*, like that of *NbBI-2*, was also significantly upregulated upon m3875 overexpression (Figure 6C). Consistent with a close phylogenetic relationship between NbWRKY9 and AtWRKY33 [57], BLAST searches of the Arabidopsis database showed that NbWRKY9 shared the highest identity with AtWRKY33 (Genbank No. NP_181381.2), suggesting that NbWRKY9 is an AtWRKY33 ortholog in *N. benthamiana*. AtWRKY33 is a well-documented WRKY transcription factor that negatively regulates defense responses mediated by salicylic acid (SA), a critical signaling molecule responsible for triggering PCD [58]. In particular, the *Arabidopsis thaliana* plants overexpressing AtWRKY33 confer high susceptibility to *Pseudomonas syringae*, a biotrophic bacterium [59].

## 3. Discussion

Bacterial pathogens often secrete proteins (also called effector proteins) that contribute to disease pathogenesis [60]. As a Gram-negative bacterium, CLas does not possess secretion systems T3SS, T4SS and T6SS, but it has the Sec secretion system [26]. To date, a total of 86 CLas-encoded proteins have been determined as Sec-dependent secretory proteins [27]. Although evidence implicates these secreted proteins as being involved in CLas pathogenesis [22,27,34,61,62], information about the contributions of these proteins to the virulence of CLas is limited. In this study, we identified a new Sec-dependent secreted protein (CLIBASIA_03875) from CLas. The transient overexpression of m3875, the mature form of CLIBASIA_03875, completely suppressed INF1- and BAX-triggered HR PCD, as well as induced dwarfing, leaf deformation and mosaics in *N. benthamiana*. This is reminiscent of a recent study in which *CLIBASIA_03875* was predicted to encode an effector protein and expressed relatively high mRNA levels in both the leaf and root tissues of Duncan and Cleopatra, two citrus species that differ in their tolerance to HLB [33]. This study, along with our current data, strongly indicates the critical role of CLIBASIA 03875 in CLas pathogenesis.

The plant HR PCD is initiated as a strong immune response to block the infection of biotrophic/hemibiotrophic pathogens [63]. Conversely, to enable a successful infection, the biotrophs and hemibiotrophs often evolve suites of PCD-suppressing effectors to impede the plant immune response. For example, the majority of the type III secretion proteins produced by the biotrophic bacterium *P. syringae* pv. *tomato* DC3000 are devoted to suppressing the host HR PCD [64,65,66]. In addition, hemibiotrophic *Phytophthora species* usually express a significant number of effectors to inhibit PCD before switching to a necrotrophic infection phase, thereby facilitating the initial infection in living host cells [45,67]. In this study, the secreted protein m3875 was identified to be a PCD suppressor, suggesting that the obligate biotroph CLas utilizes the tactic frequently employed by the biotrophic/hemibiotrophic pathogens to prevent the plant HR PCD.

The transient overexpression of m3875 in *N. benthamiana* regulates the transcriptional levels of a range of defense-related genes, including *NbCNGC23*-*26*, *NbBI-2* and *Nbwrky9*. *NbCNGC23*-*26* was of particular interest, since this gene is essential for conducting Ca^2+^ into the cytosol. Ca^2+^ is a key regulator that contributes to triggering the HR PCD and ensures its timely and controlled execution. An iIncrease in Ca^2+^ influx is therefore an initial protective response following pathogen detection [37,48]. Previous studies have delineated that the effectors typically suppress PCD via targeting pathogen perception, ROS signaling networks, or mitogen-activated protein kinase (MAPK) cascades [7,68]. However, there is little information regarding the mechanism by which PCD-suppressing effectors can interfere with Ca^2+^ influx. In this study, we detected that m3875 caused a significant reduction in the expression of *NbCNGC23*-*26*, which are all CNGCs group-IVb members of *N. benthamiana*. It has long been known that the CNGCs group-IVb members of *A. thaliana* (AtCNGC2 and AtCNGC4) are positive regulators of HR [52,53]. Taken together, we present a novel PCD-suppressing pathway in which m3875 indirectly reduces calcium influx by reducing the expression of *NbCNGC23*-*26* to ultimately suppress PCD.

In contrast to *NbCNGC23*-*26*, *NbWRKY9* and *NbBI-2* were significantly upregulated during the transient expression of m3875. NbWRKY9 is required to activate the RBOH (respiratory burst oxidase homologue)**-dependent ROS burst in *N. benthamiana* [57]. The ectopic overexpression of *AtWRKY33*, the closest Arabidopsis WRKY to NbWRKY9, in *A. thaliana* has been shown to improve disease resistance to the necrotrophic fungi *Botrytis cinerea* and *Alternaria brassicicola* [58]. However, the AtWRKY33-overexpressing plants exhibited increased susceptibility to the biotrophic bacterium *P. syringae* [58]. Similarly, the overexpression of *HvBI-1* in barley increases resistance to the necrotrophic fungus *Fusarium graminearum* [69] but confers increased susceptibility to the biotrophic fungus *Blumeria graminis* f. sp. *hordei* [69,70,71,72]. These observations suggest that both WRKY33 and BI- 1 act as so-called susceptibility (S) factors for biotrophs [73]. In addition, BI-1 is a well-known PCD suppressor [55], although BI-1 acts to trigger cell death when transiently overexpressed under the control of a double *35S* promoter and a translation enhancer TMV Omega leader sequence [56]. Therefore, the m3875-enhanced expression of *NbWRKY9* and *NbBI-2* may reduce the host innate immune response against biotrophs, indicating a potential strategy for CLas infection. Notably, m3875 did not alter the expression of *NbBI-1*, an additional BI-1 homolog in *N. benthamiana* known to interact with ATG6 to regulate autophagy and PCD [56], suggesting that NbBI-1 and NbBI-2 have different roles in the susceptibility to the biotrophic pathogen.

The putative m3875 is a small protein comprising only 22 amino acids. However, site-directed mutagenesis of the charged amino acid (D^7^, R^9^, R^11^ or K^22^) or even the simultaneous substitution of D7, R9, and R11 with alanine residues within m3875 had little effect on its functions in PCD suppression and plant development. While there have been repeated observations that proteins exist with robustness against site mutations, without conferring loss of function [74,75], the current data are still surprising considering the small size of m3875. Previous studies have elucidated that this mutational robustness promotes the evolution of protein [76,77], resulting in more robust proteins with a greater capacity to acquire new or improved functions over large evolutionary time-scales [78]. In view of the positive correlation between mutational robustness and evolvability [79,80], it is plausible that the robustness of m3875 would facilitate its evolvability, thereby offering benefits to CLas for its survival and colonization in host plants.

Collectively, in this study, the mature form of a new CLas-encoded Sec-dependent secretory protein was shown to inhibit HR PCD formation in *N. benthamiana*, and the functional mechanisms behind this interaction were preliminarily investigated. It is known that CLas infects all commercial citrus species and scion cultivars regardless of their rootstock [17,18]. Unfortunately, the underlying mechanisms regarding how this bacterium overwhelms plant immune responses remain elusive. In this study, the identification of m3875 as the PCD suppressor suggests a novel strategy of CLas to antagonize the host innate immunity, which may extend the current understanding of CLas virulence mechanisms, as well as host plant susceptibility, and therefore, merits further investigation.

## 4. Materials and Methods

### 4.1. Plants, Microbial Strains, and Growth Conditions

The *N. benthamiana* plants were grown in a greenhouse at room temperature with a photoperiod of 18 h light/6 h darkness. *Escherichia coli* strains DH5α and BL21 were grown on Luria-Bertani (LB) medium at 37 °C, while *Agrobacterium tumefaciens* strains EHA105 and GV3101 were on Yeast Extract Broth (YEB) supplemented with 50 µg/mL rifampicin, 50 µg/mL kanamycin, and 2 µg/mL tetracycline as needed.

### 4.2. In Silico Analysis of the Signal Peptide

The putative SP of CLIBASIA_03875 was predicted using the online algorithms of PrediSi [31] and SignalP version 3.0 [32] with default settings for Gram-negative bacteria.

### 4.3. Alkaline Phosphatase (PhoA) Assay

A PhoA assay system [34] was utilized to verify the putative SP of CLIBASIA_03875. Briefly, the gene encoding CLIBASIA_03875 was cloned with the primer pair 3875F/3875R (Supplementary Table S1) and inserted into vector pMD18-T (TaKaRa, Kusatsu, Japan) to generate pMD-3875. Using pMD-3875 as a template, PCR was performed with the primers 3875SP-F/3875SP-R (Supplementary Table S1). The PCR amplified the DNA sequence of the CLIBASIA_03875 N-terminal 35 amino acids, which contained the putative SP, followed by six more downstream residues. The resulting PCR product was ligated into *Nde*I-*Hin*dIII-digested pET-mphoA [34], generating a construct pET- 3875SP- mphoA containing an in-frame gene fusion between the *SP* and *mphoA* genes. The pET- 3875SP-mphoA was then introduced into *E. coli* BL21 chemically competent cells, followed by the detection of the PhoA activity of the transformants on indicator LB agar with 90 µg/mL BCIP, 100 mM IPTG and 75 mM Na_2_HPO_4_. The *E. coli* BL21 cells with the pET-phoA plasmid were used as a positive control, while the negative control was the BL21 cells that harbored pET-mphoA. After 6–10 h incubation at 37 °C, the change to blue color of the transformants indicates PhoA activity, while the colonies that remained white were deemed to lack PhoA.

### 4.4. Subcellular Localization of m3875 in Plant Cells

The sequence of m3875 was amplified using the primers m3875gfp-F/m3875gfp-R (Supplementary Table S1) and ligated into *Kpn*I-*Xho*I-digested pCAMBIA1300-35S-GFP. The resulting construct pm3875-GFP could express the C-terminal GFP fusion protein m3875-GFP. This construct was then transformed into *A. tumefaciens* EHA105, followed by the agroinfiltration of 4- week-old *N. benthamiana* as previously described [34]. GFP fluorescence of the infiltrated leaves was visualized at 60 h post inoculation (hpi) with a LSM700 confocal microscope (Zeiss, Munich, Germany).

### 4.5. Agrobacterium-Mediated PVX Infection Assay

The DNA fragments encoding m3875 were amplified with the primers m3875-F/m3875-R (Supplementary Table S1) and inserted into *Cla*I-*Sal*I-digested pGR107, a binary plant expression vector based on PVX [28], resulting in the production of pPVX-m3875. In addition, four alanine- substitution m3875 mutants were individually generated by direct PCR amplification with the corresponding primers (Supplementary Table S1) and ligated into pGR107 to generate pPVX-A7, pPVX-A9, pPVX-A11, pPVXA7-11, and pPVX-A22, respectively. These constructs were then transformed into *A. tumefaciens* GV3101.

The PCD suppression assay was performed as previously described [45]. Briefly, *A. tumefaciens* cells harboring pPVX-m3875, pPVX-A7, pPVX-A9, pPVX-A11, pPVXA7-11, or pPVX-A22 were first infiltrated into the newly expanded leaves of six *N. benthamiana* plants at the six- to seven-leaf stage. A negative control of *A. tumefaciens* cells harboring pPVX-GFP was used. At 24 hpi, the initially infiltrated sites were challenged with *A. tumefaciens* cells harboring the pGR-Bax or pGR-INF1. After 72 hpi, one third of the infiltrated leaves were detached from the plants to detect H_2_O_2_ accumulation with 3,3′-diaminobenzidine (DAB) staining as previously described [81], while the remaining leaves were observed for up to 5 days dpi to record the symptoms of cell death. The experiment was repeated three times.

To further determine the virulence of m3875, *A. tumefaciens* cells harboring pPVX-m3875 or the alanine-substitution m3875 mutants were infiltrated into six *N. benthamiana* seedlings at the three- to four-leaf stage, as previously described [28]. *A. tumefaciens* cells harboring empty pGR107 were used as a control. At 15 dpi, the systemic symptoms of the infiltrated plants were recorded, and the systemically infected leaves at a similar developmental stage were harvested to prepare the total RNA. A Northern blot analysis was subsequently performed to detect viral RNAs using a DIG-labeling RNA probe complementary to the PVX coat protein-encoding gene (GenBank No. NC_011620) as previously described [34]. The intensities of the RNA bands were quantified using Image J Version 1.45S (NIH, Bethesda, MD, USA). The level of accumulation of each construct was quantified as the ratio of the intensity of the viral gRNA to that of the corresponding 28S rRNA and normalized to that of PVX. Each construct was evaluated in three independent experiments.

### 4.6. RT-qPCR Analysis

*A. tumefaciens* cells harboring pPVX-m3875 or pPVX-eGFP were infiltrated into the fourth and fifth leaves of six *N. benthamiana* plants at the six-leaf stage. At 48 hpi, the infiltrated leaf tissues were collected and pooled for total RNA extraction using the RNeasy Mini Kit (Qiagen, Valencia, CA, USA) according to the manufacturer’s instructions. Immediately, total RNA was reverse-transcribed into first strand cDNA using the PrimeScript TM RT reagent Kit (TaKaRa, Kusatsu, Japan) with oligo (dT) as the primer. qPCR was performed on an ABI StepOne plus Real-time PCR instrument (Applied Biosystems, Foster City, CA, USA) with a 20 μL reaction system containing 5 μL of 2 X SYBR Premix Ex Taq (TaKaRa), 2 μmol/L of each gene-specific primers (listed in Supplementary Table S1), 0.5 μL of the cDNA sample, and 0.2 μL of Rox Reference Dye II (TaKaRa, Kusatsu, Japan). The reactions were performed using the following program: 95 °C for 5 min, 40 cycles of 95 °C for 15 s, 55 °C for 30 s, and 72 °C for 30 s. The *N. benthamiana* actin gene (Genbank No. JQ256516.1) was used as an internal reference. The experiment was performed with three biological replicates (each consisting of three technical replicates). Finally, the 2^-ΔΔCt^ method [82] was utilized to calculate the relative gene expression values, which were subsequently transformed to fold-change and plotted in the figures. Statistical analyses of all data were conducted using the Student’s *t*-test (SPSS 10.0, Chicago, IL, USA).

## Figures and Tables

**Table 1:** Characteristics of the alanine-substituted m3875 mutants in programmed cell death suppression.

Mutant ^a^	PCD Inducer ^b^	
	BAX	INF1
m3875	0/74	0/78
GFP	74/74	78/78
A7	0/32	2/33
A9	0/32	2/33
A11	3/32	4/34
A7-11	0/30	3/35
A22	1/32	0/35

^a^ The *Agrobacterium tumefaciens* cells harboring pPVX-m3875 or the alanine-substituted m3875 mutants were infiltrated into *Nicotiana benthamiana* as described in the Materials and Methods. Results of three separate experiments were combined. ^b^ The programmed cell death (PCD) inducers included the pro-apoptotic mouse BAX and the *Phytophthora infestans* elicitin INF1. Symptoms of cell death were observed up to 5 days post inoculation (dpi). The PCD suppression of the wild-type (WT) m3875 and its corresponding mutants are expressed as ‘lesion number/infiltration number’. No significant difference was detected between the WT m3875 and any of the five mutants (Student’s t-test, *p* > 0.05).

## Supplementary Materials

The supplementary materials can be found at https://www.mdpi.com/1422-0067/20/22/5802/s1.

## References

[1] Jones J.D.G., Dangl J.L. (2006). The plant immune system. Nature.

[2] Boller T., Felix G. (2009). A renaissance of elicitors: Perception of microbe-associated molecular patterns and danger signals by pattern-recognition receptors. Annu. Rev. Plant Biol..

[3] De Wit P.J. (2007). How plants recognize pathogens and defend themselves. Cell. Mol. Life Sci..

[4] Zvereva A.S., Golyaev V., Turco S., Gubaeva E.G., Rajeswaran R., Schepetilnikov M.V., Srour O., Ryabova L.A., Boller T., Pooggin M.M. (2016). Viral protein suppresses oxidative burst and salicylic acid-dependent autophagy and facilitates bacterial growth on virus-infected plants. New Phytol..

[5] Da Cunha L., Sreerekha M.V., Mackey D. (2007). Defense suppression by virulence effectors of bacterial phytopathogens. Curr. Opin. Plant Biol..

[6] Lo Presti L., Lanver D., Schweizer G., Tanaka S., Liang L., Tollot M., Zuccaro A., Reissmann S., Kahmann R. (2015). Fungal effectors and plant susceptibility. Annu. Rev. Plant Biol..

[7] Dou D., Zhou J.M. (2012). Phytopathogen effectors subverting host immunity: Different foes, similar battleground. Cell Host Microbe.

[8] Goverse A., Smant G. (2014). The activation and suppression of plant innate immunity by parasitic nematodes. Annu. Rev. Phytopathol..

[9] Deslandes L., Rivas S. (2012). Catch me if you can: bacterial effectors and plant targets. Trends Plant Sci..

[10] Macho A.P., Zipfel C. (2015). Targeting of plant pattern recognition receptor-triggered immunity by bacterial type-III secretion system effectors. Curr. Opin. Microbiol..

[11] Bové J.M. (2006). Huanglongbing: A destructive, newly-emerging, century-old disease of citrus. J. Plant Pathol..

[12] Gottwald T.R. (2010). Current epidemiological understanding of citrus Huanglongbing. Annu. Rev. Phytopathol..

[13] Da Graça J.V., Douhan G.W., Halbert S.E., Keremane M.L., Lee R.F., Vidalakis G., Zhao H. (2015). Huanglongbing: An overview of a complex pathosystem ravaging the world’s citrus. J. Integr. Plant Biol..

[14] Wang N., Pierson E.A., Setubal J.C., Xu J., Levy J.G., Zhang Y., Li J., Rangel L.T., Martins J. (2017). The Candidatus Liberibacter–host interface: Insights into pathogenesis mechanisms and disease control. Annu. Rev. Phytopathol..

[15] Zheng Z., Chen J., Deng X. (2018). Historical perspectives, management, and current research of citrus HLB in Guangdong Province of China, where the disease has been endemic for over a hundred years. Phytopathology.

[16] Bové J.M., Ayres A.J. (2007). Etiology of three recent diseases of citrus in Sao Paulo State: Sudden death, variegated chlorosis and huanglongbing. IUBMB Life.

[17] Folimonova S.Y., Robertson C.J., Garnsey S.M., Gowda S., Dawson W.O. (2009). Examination of the responses of different genotypes of citrus to huanglongbing (citrus greening) under different conditions. Phytopathology.

[18] Martinelli F., Dandekar A.M. (2017). Genetic mechanisms of the devious intruder *Candidatus Liberibacter* in citrus. Front. Plant Sci..

[19] Jain M., Fleites L.A., Gabriel D.W. (2015). Prophage-encoded peroxidase in ‘*Candidatus* Liberibacter asiaticus’ is a secreted effector that suppresses plant defenses. Mol. Plant Microbe Interact..

[20] Jain M., Munoz-Bodna A., Zhang S., Gabriel D.W. (2018). A secreted ‘*Candidatus* Liberibacter asiaticus’ peroxiredoxin simultaneously suppresses both localized and systemic innate immune responses in planta. Mol. Plant Microbe Interact..

[21] Jain M., Munoz-Bodnar A., Gabriel D.W. (2019). ‘*Candidatus* Liberibacter asiaticus’ peroxiredoxin (LasBCP) suppresses oxylipin-mediated defense signaling in citrus. J. Plant Physiol..

[22] Clark K., Franco J.Y., Schwizer S., Pang Z., Hawara E., Liebrand T.W.H., Pagliaccia D., Zeng L., Gurung F.B., Wang P. (2018). An effector from the Huanglongbing-associated pathogen targets citrus proteases. Nat. Commun..

[23] Cambronne E.D., Roy C.R. (2006). Recognition and delivery of effector proteins into eukaryotic cells by bacterial secretion systems. Traffic.

[24] Shames S.R., Finlay B.B. (2012). Bacterial effector interplay: A new way to view effector function. Trends Microbiol..

[25] Galán J.E., Waksman G. (2018). Protein-injection machines in bacteria. Cell.

[26] Duan Y., Zhou L., Hall D.G., Li W., Doddapaneni H., Lin H., Liu L., Vahling C.M., Gabriel D.W., Williams K.P. (2009). Complete genome sequence of citrus Huanglongbing bacterium, ‘*Candidatus* Liberibacter asiaticus’ obtained through metagenomics. Mol. Plant Microbe Interact..

[27] Prasad S., Xu J., Zhang Y., Wang N. (2016). SEC-translocon dependent extracytoplasmic proteins of *Candidatus Liberibacter* asiaticus. Front. Microbiol..

[28] Jones L., Hamilton A.J., Voinnet O., Thomas C.L., Maule A.J., Baulcombe D.C. (1999). RNA-DNA interactions and DNA methylation in post-transcriptional gene silencing. Plant Cell.

[29] Katoh H., Miyata S., Inoue H., Iwanami T. (2014). Unique features of a Japanese ‘*Candidatus* Liberibacter asiaticus’ strain revealed by whole genome sequencing. PLoS ONE.

[30] Teixeira D.C., Saillard C., Eveillard S., Danet J.L., da Costa P.I., Ayres A.J., Bové J. (2005). “*Candidatus* Liberibacter americanus”, associated with citrus Huanglongbing (greening disease) in São Paulo state, Brazil. Int. J. Syst. Evol. Microbiol..

[31] Hiller K., Grote A., Scheer M., Münch R., Jahn D. (2004). PrediSi: Prediction of signal peptides and their cleavage positions. Nucleic Acids Res..

[32] Bendtsen J.D., Nielsen H., von Heijne G., Brunak S. (2004). Improved prediction of signal peptides: SignalP 3.0. J. Mol. Biol..

[33] Shi Q., Pitino M., Zhang S., Krystel J., Cano L.M., Shatters R.G., Hall D.G., Stover E. (2019). Temporal and spatial detection of *Candidatus* Liberibacter asiaticus putative effector transcripts during interaction with Huanglongbing-susceptible, -tolerant, and -resistant citrus hosts. BMC Plant Biol..

[34] Liu X., Fan Y., Zhang C., Dai M., Wang X., Li W. (2019). Nuclear import of a secreted ‘*Candidatus Liberibacter asiaticus*’ protein is temperature dependent and contributes to pathogenicity in *Nicotiana benthamiana*. Front. Microbiol..

[35] Cranford-Smith T., Huber D. (2018). The way is the goal: How SecA transports proteins across the cytoplasmic membrane in bacteria. FEMS Microbiol. Lett..

[36] Van der Hoorn R.A., Laurent F., Roth R., De Wit P.J. (2000). Agroinfiltration is a versatile tool that facilitates comparative analyses of Avr9/Cf-9-induced and Avr4/Cf-4-induced necrosis. Mol. Plant Microbe Interact..

[37] Huysmans M., Lema A.S., Coll N.S., Nowack M.K. (2017). Dying two deaths—Programmed cell death regulation in development and disease. Curr. Opin. Plant Biol..

[38] Stael S., Kmiecik P., Willems P., Van Der Kelen K., Coll N.S., Teige M., Van Breusegem F. (2015). Plant innate immunity-sunny side up?. Trends Plant Sci..

[39] Lacomme C., Santa Cruz S. (1999). Bax-induced cell death in tobacco is similar to the hypersensitive response. Proc. Natl. Acad. Sci. USA.

[40] Baek D., Nam J., Koo Y.D., Kim D.H., Lee J., Jeong J.C., Kwak S.S., Chung W.S., Lim C.O., Bahk J.D. (2019). Bax-induced cell death of Arabidopsis is meditated through reactive oxygen-dependent and -independent processes. Plant Mol. Biol..

[41] Kamoun S., van West P., Vleeshouwers V.G., de Groot K.E., Govers F. (1998). Resistance of *Nicotiana benthamiana* to *Phytophthora infestans* is mediated by the recognition of the elicitor protein INF1. Plant Cell.

[42] Li Z., Yin Z., Fan Y., Xu M., Kang Z., Huang L. (2015). Candidate effector proteins of the necrotrophic apple canker pathogen *Valsa mali* can suppress BAX-induced PCD. Front. Plant Sci..

[43] Fang A., Gao H., Zhang N., Zheng X., Qiu S., Li Y., Zhou S., Cui F., Sun W. (2019). A novel effector gene SCRE2 contributes to full virulence of *Ustilaginoidea virens* to rice. Front Microbiol..

[44] Bos J.I., Kanneganti T.D., Young C., Cakir C., Huitema E., Win J., Armstrong M.R., Birch P.R., Kamoun S. (2006). The C-terminal half of *Phytophthora infestans* RXLR effector AVR3a is sufficient to trigger R3a-mediated hypersensitivity and suppress INF1-induced cell death in *Nicotiana benthamiana*. Plant J..

[45] Wang Q., Han C., Ferreira A.O., Yu X., Ye W., Tripathy S., Kale S.D., Gu B., Sheng Y., Sui Y. (2011). Transcriptional programming and functional interactions within the *Phytophthora sojae* RXLR effector repertoire. Plant Cell.

[46] Zhuo K., Chen J., Lin B., Wang J., Sun F., Hu L., Liao J. (2017). A novel *Meloidogyne enterolobii* effector MeTCTP promotes parasitism by suppressing programmed cell death in host plants. Mol. Plant Pathol..

[47] Petrov V.D., Van Breusegem F. (2012). Hydrogen peroxide-a central hub for information flow in plant cells. AoB Plants.

[48] Yuan P., Jauregui E., Du L., Tanaka K., Poovaiah B.W. (2017). Calcium signatures and signaling events orchestrate plant–microbe interactions. Curr. Opin. Plant Biol..

[49] Demidchik V., Shabala S., Isayenkov S., Cuin T.A., Pottosin I. (2018). Calcium transport across plant membranes: Mechanisms and functions. New Phytol..

[50] Jammes F., Hu H.C., Villiers F., Bouten R., Kwak J.M. (2011). Calcium-permeable channels in plant cells. FEBS J..

[51] Mäser P., Thomine S., Schroeder J.I., Ward J.M., Hirschi K., Sze H., Talke I.N., Amtmann A., Maathuis F.J., Sanders D. (2001). Phylogenetic relationships within cation transporter families of *Arabidopsis*. Plant Physiol..

[52] Balagué C., Lin B., Alcon C., Flottes G., Malmström S., Köhler C., Neuhaus G., Pelletier G., Gaymard F., Roby D. (2003). HLM1, an essential signaling component in the hypersensitive response, is a member of the cyclic nucleotide-gated channel ion channel family. Plant Cell.

[53] Clough S.J., Fengler K.A., Yu I.C., Lippok B., Smith R.K., Bent A.F. (2000). The *Arabidopsis* dnd1 “defense, no death” gene encodes a mutated cyclic nucleotide-gated ion channel. Proc. Natl. Acad. Sci. USA.

[54] Nawaz Z., Kakar K.U., Ullah R., Yu S., Zhang J., Shu Q.Y., Ren X.L. (2019). Genome-wide identification, evolution and expression analysis of cyclicnucleotide-gated channels in tobacco (*Nicotiana tabacum* L.). Genomics.

[55] Ishikawa T., Watanabe N., Nagano M., Kawai-Yamada M., Lam E. (2011). Bax inhibitor-1: A highly conserved endoplasmic reticulum-resident cell death suppressor. Cell Death Differ..

[56] Xu G., Wang S., Han S., Xie K., Wang Y., Li J., Liu Y. (2017). Plant bax inhibitor-1 interacts with ATG6 to regulate autophagy and programmed cell death. Autophagy.

[57] Adachi H., Nakano T., Miyagawa N., Ishihama N., Yoshioka M., Katou Y., Yaeno T., Shirasu K., Yoshioka H. (2015). WRKY transcription factors phosphorylated by MAPK regulate a plant immune NADPH Oxidase in *Nicotiana benthamiana*. Plant Cell.

[58] Lorrain S., Vailleau F., Balagué C., Roby D. (2003). Lesion mimic mutants: Keys for deciphering cell death and defense pathways in plants?. Trends Plant Sci..

[59] Zheng Z., Qamar S.A., Chen Z., Mengiste T. (2006). Arabidopsis WRKY33 transcription factor is required for resistance to necrotrophic fungal pathogens. Plant J..

[60] Stavrinides J., McCann H.C., Guttman D.S. (2008). Host-pathogen interplay and the evolution of bacterial effectors. Cell Microbiol..

[61] Pitino M., Allen V., Duan Y. (2018). LasΔ5315 Effector induces extreme starch accumulation and chlorosis as Ca. Liberibacter asiaticus infection in *Nicotiana benthamiana*. Front. Plant Sci..

[62] Pitino M., Armstrong C.M., Cano L.M., Duan Y. (2016). Transient expression of *Candidatus* Liberibacter asiaticus effector induces cell death in *Nicotiana benthamiana*. Front. Plant Sci..

[63] Glazebrook J. (2005). Contrasting mechanisms of defense against biotrophic and necrotrophic pathogens. Annu. Rev. Phytopathol..

[64] Jamir Y., Guo M., Oh H.S., Petnicki-Ocwieja T., Chen S., Tang X., Dickman M.B., Collmer A., Alfano J.R. (2004). Identification of *Pseudomonas syringae* type III effectors that can suppress programmed cell death in plants and yeast. Plant J..

[65] Guo M., Tian F., Wamboldt Y., Alfano J.R. (2009). The majority of the type III effector inventory of *Pseudomonas syringae* pv. *tomato* DC3000 can suppress plant immunity. Mol. Plant Microbe Interact..

[66] Wei H.L., Zhang W., Collmer A. (2018). Modular study of the type III effector repertoire in *Pseudomonas syringae* pv. *tomato* DC3000 reveals a matrix of effector interplay in pathogenesis. Cell Rep..

[67] Pais M., Win J., Yoshida K., Etherington G.J., Cano L.M., Raffaele S., Banfield M.J., Jones A., Kamoun S., Saunders D.G. (2013). From pathogen genomes to host plant processes: The power of plant parasitic oomycetes. Genome Biol..

[68] Mukhtar M.S., McCormack M.E., Argueso C.T., Pajerowska-Mukhtar K.M. (2016). pathogen tactics to manipulate plant cell death. Curr. Biol..

[69] Babaeizad V., Imani J., Kogel K.H., Eichmann R., Hückelhoven R. (2009). Over-expression of the cell death regulator BAX inhibitor-1 in barley confers reduced or enhanced susceptibility to distinct fungal pathogens. Theor. Appl. Genet..

[70] Hückelhoven R., Dechert C., Kogel K.H. (2003). Overexpression of barley BAX inhibitor 1 induces breakdown of mlo-mediated penetration resistance to *Blumeria graminis*. Proc. Natl. Acad. Sci. USA.

[71] Eichmann R., Schultheiss H., Kogel K.H., Hückelhoven R. (2004). The barley apoptosis suppressor homologue BAX inhibitor-1 compromises nonhost penetration resistance of barley to the inappropriate pathogen *Blumeria graminis* f. sp. *tritici*. Mol. Plant Microbe Interact..

[72] Eichmann R., Bischof M., Weis C., Shaw J., Lacomme C., Schweizer P., Duchkov D., Hensel G., Kumlehn J., Hückelhoven R. (2010). BAX INHIBITOR-1 is required for full susceptibility of barley to powdery mildew. Mol. Plant Microbe Interact..

[73] Van Schie C.C.N., Takken F.L.W. (2014). Susceptibility genes 101: How to be a good host. Annu. Rev. Phytopathol..

[74] Taverna D.M., Goldstein R.A. (2002). Why are proteins so robust to site mutations?. J. Mol. Biol..

[75] Guo H.H., Choe J., Loeb L.A. (2004). Protein tolerance to random amino acid change. Proc. Natl. Acad. Sci. USA.

[76] Bershtein S., Segal M., Bekerman R., Tokuriki N., Tawfifik D.S. (2006). Robustness-epistasis link shapes the fifitness landscape of a randomly drifting protein. Nature.

[77] Bloom J.D., Labthavikul S.T., Otey C.R., Arnold F.H. (2006). Protein stability promotes evolvability. Proc. Natl. Acad. Sci. USA.

[78] Ferrada E., Wagner A. (2008). Protein robustness promotes evolutionary innovations on large evolutionary time-scales. Proc. Biol. Sci..

[79] Tokuriki N., Tawfik D.S. (2009). Stability effects of mutations and protein evolvability. Curr. Opin. Struct. Biol..

[80] Tóth-Petróczy A., Tawfik D.S. (2014). The robustness and innovability of protein folds. Curr. Opin. Struct. Biol..

[81] Thordal-Christensen H., Zhang Z., Wei Y., Collinge D.B. (1997). Subcellular localization of H_2_O_2_ in plants. H_2_O_2_ accumulation in papillae and hypersensitive response during the barley-powdery mildew interaction. Plant J..

[82] Livak K.J., Schmittgen T.D. (2001). Analysis of relative gene expression data using real-time quantitative PCR and the 2 (-Delta Delta C (T)) method. Methods.

