# Supplementary material for: A Novel ‘Candidatus Liberibacter asiaticus’-Encoded Sec-Dependent Secretory Protein Suppresses Programmed Cell Death in Nicotiana benthamiana
Source: Int J Mol Sci. 2019 Nov 18;20(22):5802. doi: 10.3390/ijms20225802 (PMC6888338; doi:10.3390/ijms20225802)
Supplement: Supplementary file 1 [file ijms-20-05802-s001.pdf]

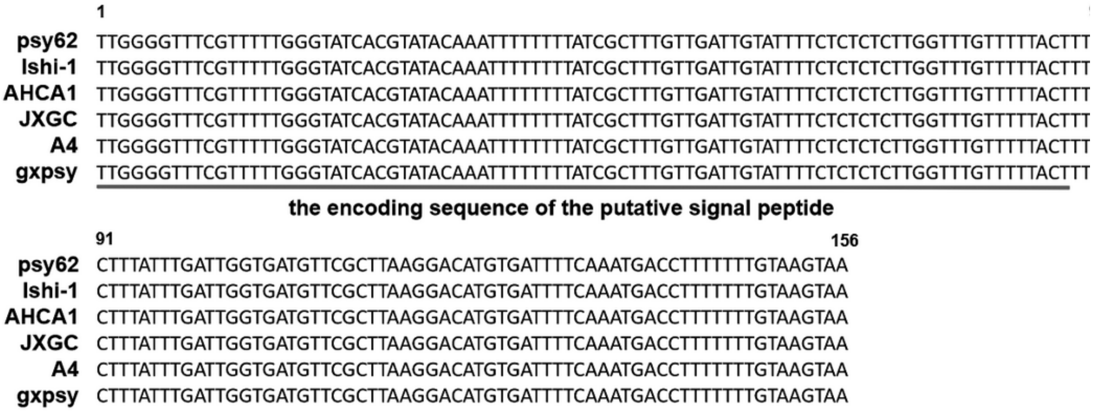

FIGURE S1. ClustalW alignment of the nucleotide sequences of CLIBASIA_03875 from six CLas strains for which the complete genome or chromosome was available, including psy62 (NC_012985.3), lshi-1 (NZ_AP014595.1), AHCA1 (NZ_CP029348.1), JXGC (NZ_CP019958.1), A4 (NZ_CP010804.1) and gxpsy (NC_020549.1). The coding sequence of the putative signal peptide is underlined.

Table S1. The primers used in this study[1]

Construct/Gene Name	Primer Name	Primer Sequence (5′-3′)
pMD3875	3875F	TTGGGGTTTCGTTTTTGGGTA
	3875R	TTACTTACAAAAAAAGGTCATTTG
pET-3875SP-mphoA	3875SP-F	AAGGAGATATA**CATATG**TTGGGGTTTCGTTTTTGGGTA
	3875SP-R	AGGCATTTCTGG**AAGCTT**ATAAAGCAAAGTAAAAACAAACCA
pm3875-GFP	m3875gfp-F	GACGAGCTCG**GGTACC**ATGTTGCTTTATTTGATTGGTGA
	m3875gfp-R	TGGTGTCGAC**TCTAGA**CTTACAAAAAAAGGTCATTTGA
pPVX-m3875	m3875-F	GAGCACCAGCTAGC**ATCGAT**ATGTTGCTTTATTTGATTGG
	m3875-R	CAAGCTTATCGGCG**GTCGAC**TTACTTACAAAAAAAGGTCATTT
pPVX-eGFP	eGFP-F	GAGCACCAGCTAGC**ATCGAT**ATGGTGAGCAAGGGCGAGGA
	eGFP-R	CAAGCTTATCGGCG**GTCGAC**TCAAAGATCTACCATGTACA
pPVX-A7	ES34m1-F	GAGCACCAGCTAGC**ATCGAT**ATGTTGCTTTATTTGATTGGTGCTGT
	m3875-R	--
pPVX-A9	ES34m2-F	GAGCACCAGCTAGC**ATCGAT**ATGTTGCTTTATTTGATTGGTGATGTT GCCTTAAG
pPVX-A11	m3875-R	--
	ES34m3-F	GAGCACCAGCTAGC**ATCGAT**ATGTTGCTTTATTTGATTGGTGATGTT CGCTTAGCG
pPVX-A7-11	m3875-R	
	ES34m4-F	GAGCACCAGCTAGCATCGATATGTTGCTTTATTTGATTGGTGCTGTT GCCTTAGC
pPVX-A22	m3875-F	--
	ES34m5-R	CAAGCTTATCGGCG**GTCGAC**TTACGCACAAAAAAAGGTCATT
**qPCR assay**		
Nbactin	NbActin-F	GCAGGAATCCACGAGACTACA
	NbActin-R	AACCTCCAATCCAGACACTGT
NbCNGC23	NbCNGC23-F	CAGTTATCGTCACGGTGATAC
	NbCNGC23-R	CCTGAGGGACAGGTAGGACGAC

NbCNGC24	NbCNGC24-F	AATCATCGTTCTTACTAGTGGGCT
	NbCNGC24-R	CATCCTCTGCTTGTAATCCTGTGG
NbCNGC25	NbCNGC25-F	CGTTCTTACCTCTGGGTTAAT
	NbCNGC25-R	GTTCTCTGCTTTAATTCTCGAGG
NbCNGC26	NbCNGC26-F	GAGAGGCTTAAGCGAACAGCA
	NbCNGC26-R	CCCATTCTCCATTACCTGATTCAC
NbBI-1	NbBI1-F	CCTTTTCTGGTTGCACTTTGCG
	NbBI1-R	AAAGAGGGTCAGAGCATGCTTC
NbBI-2	NbBI2-F	GCACTCTTTGAAGGGGCTTCTG
	NbBI2-R	CCCAAGGTACAAGTACTCTCTGC
NbWRKY9	NbWRKY9-F	CATCCAAAGCCTCAGTCCAC
	NbWRKY9-R	CCTTCCATCTCTTCGCCTCA

1. The restriction recognition sequences of *Nde* I (CATATG), *Hin*d III (AAGCTT), *Kpn* I (GGTACC), *Xba* I (TCTAGA), *Cla* I (ATCGAT) and *Sal* I (GTCGAC) within the primers are in bold and underlined. Genes included in qPCR assay are *Nbactin* (GenBank No. JQ256516.1), *NbBI-1* (GenBank No. JX481914.1), *NbWRKY9* (GenBank No. AB711130.1), as well as five additional ones *NbCNGC23* (Niben101Scf08863g01010.1), *NbCNGC24* (Niben101Scf08680g02015.1), *NbCNGC25* (Niben101Scf04528g09004.1), *NbCNGC26* (Niben101Scf08157g02012.1) and NbBI-2 (Niben101Scf02705g01020.1) from *N. benthamiana* draft genome sequence V1.0.1 (https://solgenomics.net/organism/Nicotiana_benthamiana/genome).
